# Integrating health intervention into existing program structure of the neglected tropical diseases: lessons learned from Yobe and Ebonyi states

**DOI:** 10.3389/fpubh.2023.1281091

**Published:** 2024-01-17

**Authors:** Sunday Atobatele, Sidney Sampson, Akolade U. Jimoh, Saheed D. Isiaka, Oluwafisayo Ayodeji, Joshua C. David, Victor Daniel, Oluwafunmilayo Dehinbo

**Affiliations:** ^1^Sydani Group, FCT, Abuja, Nigeria; ^2^Sydani Institute for Research and Innovation, Abuja, Nigeria

**Keywords:** integration, health intervention, neglected tropical diseases (NTD), community drug distributors (CDDs), service delivery and mass administration of medicine (MAM)

## Abstract

**Background:**

Nigeria has a national policy on neglected tropical diseases (NTD) which is coordinated by the Federal Ministry of Health and integrated into the health system at all government levels. A network of grassroots mobilizers, named community drug distributors (CDDs), deliver NTD drugs and commodities to rural and hard-to-reach communities throughout Nigeria.

**Methods:**

Interviews with state and local government coordinators of Nigerian NTD programs and focus group discussions with CDDs in Yobe and Ebonyi states were conducted to understand the working modalities of NTD programs in Nigeria to identify the potential of leveraging the NTD structure for additional health interventions such as COVID-19 vaccine rollouts. Collected data was coded and managed on NVivo version 12 using content analysis.

**Results and discussion:**

The study found that the NTD committee had the following: a structured planning and coordination process, a community mobilization approach for the effective delivery of routine Mass Administration of Medication (MAM) activities. Challenges encountered included little or no incentives for the CDDs, insecurity, transportation and logistics, and lack of equipment and drug commodities. Nigerian NTD health structures have a wide reach, with a presence in all states and local government areas (LGAs), and this has previously been leveraged to deliver commodities and interventions to rural and hard-to-reach communities for non-NTD health programs and could thus be used similarly for COVID-19 vaccination programs. The enablers of integrating health interventions into the NTD structure are increased availability of finances and manpower, while lack of financial incentives and partners’ buy-in were identified as possible. challenges.

**Conclusion:**

The study suggests that integrating COVID-19 interventions into the NTD program in Nigeria would be a significant accomplishment, as the existing structure can support future interventions. The study also highlights the efficiency of the NTD program’s modalities and processes, indicating that an organized structural system for public health interventions can. increase the services of existing interventions while allowing for the integration of future interventions.

## Background

There is evidence that despite the persistent debates surrounding integrated care for over four decades, its impact on population health has been nothing short of remarkable. This is particularly evident in countries where integrated care has been implemented, with notable improvements in accessibility to healthcare services and substantial enhancements in health outcomes (1, 2). For example, healthcare integration has aided quantum leaps toward achieving the goal of Universal Health Coverage (3, 4), and this has been largely driven by the fact that the idea of health integration emerged at the Alma Ata Declaration conference (5) with the ultimate aim of “*health for all*,” adopting a Bottom (Primary Health Care – PHC) – Top Approach.

Integrated care is crucial to the dynamics of the health systems of countries all over the world (6), as integrating healthcare services empowers individuals and communities to effortlessly access comprehensive and effective promotive, preventive, curative, rehabilitative, and palliative health services, without jeopardizing their financial well-being (7, 8). For example, in promoting immunization and vaccine-related issues, healthcare integration was recognized by the Global Vaccine Action Plan (GVAP) 2011–2020 as it was included as the fourth of its six guiding principles. According to GVAP, “strong immunization systems, as part of broader health systems and closely coordinated with other primary healthcare delivery programs, are essential for achieving immunization goals” (9). Also highlighted by the GVAP was that “immunization service delivery should continue to serve as a platform for providing other priority public health interventions, such as those for vitamin A supplementation, deworming, and insecticide-treated bed nets, and that other priority programs should also serve as a platform for delivering immunization,” as such, the GVAP also noted that “every contact with the health sector should be used as an opportunity to verify immunization status and provide immunization where indicated” (9).

Although immunization has been integrated with healthcare, especially at the primary healthcare level in many LMICs, including Nigeria (10), there was an evident infrastructure (cold-chain system) and manpower deficiency, especially in rural areas (11). Additionally, rural communities have been known to have poor access to, and utilization of health services, and this was more evident with the COVID-19 vaccine deployment programs as some rural and hard-to-reach communities were completely cut off from the COVID-19 vaccination programs (12). In a bid to achieve herd immunity, and improve COVID-19 vaccine uptake in Nigeria, the National Primary Healthcare Development Agency (NPHCDA), which is the coordinating agency for the vaccination programs in Nigeria, adopted multiple strategies to facilitate the deployment and administration of COVID-19 vaccines (13). One of many such strategies was leveraging the Preventive Chemotherapy for Neglected Tropical Diseases (PC-NTD) annually conducted in all the states in Nigeria in several rural communities that have been identified as hotspots where neglected tropical diseases (NTDs) such as Onchocerciasis, Schistosomiasis, among others, are deeply entrenched, posing persistent challenges for disease control and eradication efforts.

Neglected Tropical Disease (NTD) is a term that was coined as a collective word for a group of diseases (comprising 20 different types, including snakebites, scabies among others) by the WHO during a series of international workshops held between 2003 and 2005 (14). This marked the transition from a fragmented care approach for these diseases to an integrated communal-centered approach (14–16). Nigeria has a national policy on the NTD, and it is coordinated by the Federal Ministry of Health (FMoH) and integrated into the Health System strategy across all levels of government, including the local government area (LGA) level. Nigeria’s policy works in conjunction with non-governmental development organizations to develop and implement programs that will manage, control, and eradicate NTDs in the nation. This policy is premised on four strategic objectives including strengthening government ownership, advocacy, coordination, and partnerships; enhancing planning for results, resource mobilization, and financial sustainability; scaling up access to interventions, treatment, and system capacity building; and enhancing NTD monitoring and evaluation, surveillance, and operational research (17).

Although several studies have argued the need to integrate public health interventions into primary care services (1, 18–20), little has been done to explore the potential of integrating public health interventions into extant structures such as the NTD structures presently in place in Nigeria. It is against this backdrop that this study seeks to explore opportunities for exploiting NTD structure for future integrated health interventions. The objectives of the study are therefore to understand the working modalities of NTD structures in Nigeria and identify the potential of leveraging them for other health interventions.

## Methods

### Study design and study participants

Our study was conducted in Yobe and Ebonyi states between March and June 2022, and designed to leverage NTD structures to improve COVID-19 vaccination in prioritized communities in both states. Yobe and Ebonyi states were purposively selected for the intervention due to having the highest prevalence of COVID-19, and the lowest vaccination rates in the Northern and Southern regions of Nigeria, respectively. One rural LGA with the lowest vaccination rate in each senatorial district per state (as three senatorial districts make up one state) was selected for the intervention, hence the intervention was conducted in 6 LGAs across both states. The qualitative study utilized a structured Focus Group Discussion (FGD) and In-depth Interviews (IDI) within these 6 LGAs to understand the working modalities of Nigerian NTD structures and explore opportunities for integrating future health interventions into them.

The study participants include the state and local NTD coordinators, program officers and community drug distributors in both Yobe and Ebonyi states. A total of 3 Focus Group Discussions and 10 In-depth Interviews were conducted in both states. Participants were purposively selected to provide detailed information about the working modalities of the NTD program, and their perception of integrating future public health interventions into the NTD program. The FGD was conducted among the CDDs to explore the challenges and opportunities for integrating future health interventions into the extant NTD structure.

### Analytical framework

**Figure 1:**
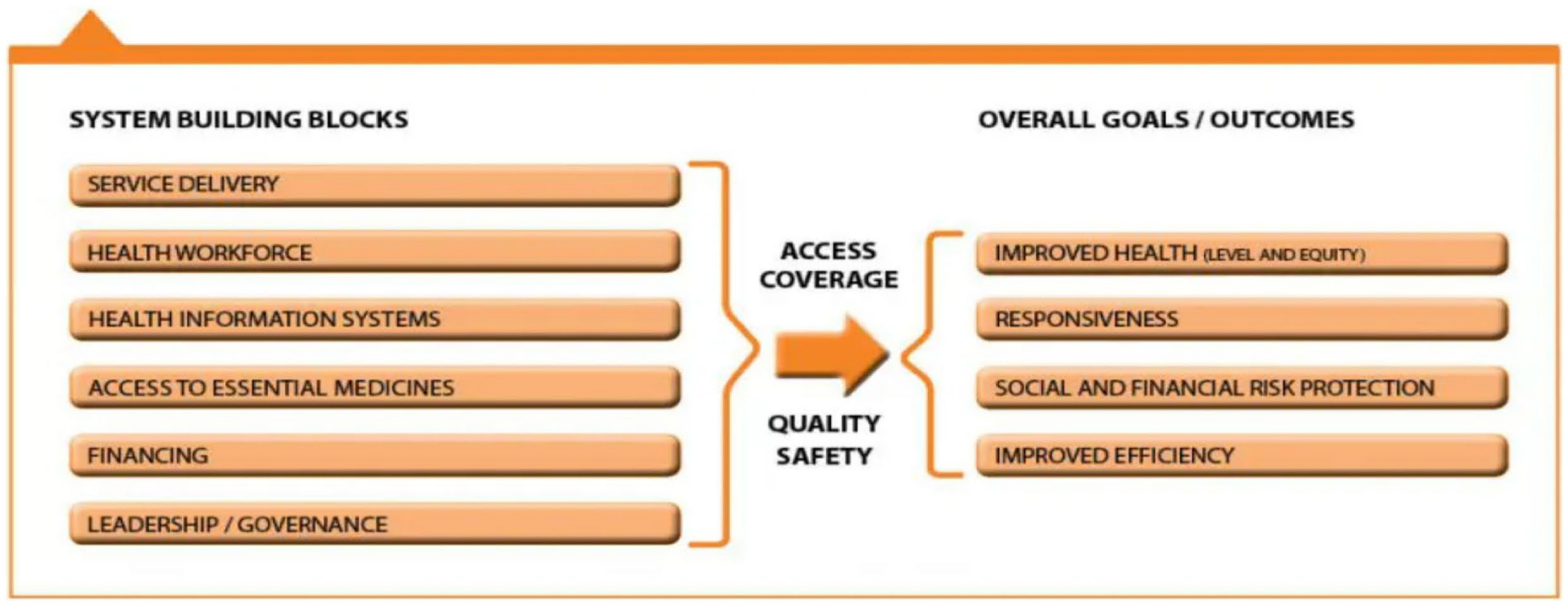
The WHO health systems framework.

This study adapted the WHO Health Systems Framework which guided our study tool design, presentation of our result, and discussion. This helps to unravel the barriers and enablers of integrating public health interventions into the Nigerian NTD structure at the community level (Figure 1).

### Data collection and analysis

Unstructured interview guides were developed for the IDI and FGD to elucidate information from the participants. The interview guides were pre-tested on a smaller population and refined with feedback, and validations.

The interviews were conducted using the interview guides, by the research assistants (RA) who had been trained on how to collect the data. Verbal consent was obtained from the participants at the start of the interviews and afterward, the discussions were conducted in English, Hausa, and Igbo languages as appropriate. None of the participants was provided with any incentive to participate in the study.

Each IDI lasted for about 45–60 min while the FGD lasted for an average of 90 and 120 min. The discussions were recorded using an audio recorder. The audio files were then translated where necessary and transcribed. The audio-recorded interviews and summary notes were translated and transcribed, while the coding and analysis were done on NVivo version 12.

### Ethical approval

The study followed the guidelines on research involving human subjects, according to the Helsinki Declaration. Written and verbal consent was obtained from the participants at the start of the IDI and FGD. Information confidentiality was guaranteed, and the discussion was anonymous. The study protocol was also approved by the National Health Research Ethics Committee (NHREC) in the Federal Ministry of Health (FMoH) Abuja: NHREC/01/01/2007–14/07/2021.

## Results

This study investigated the working modalities of the NTD structure and explored the potential of leveraging the NTD structure for other health interventions. To better understand the potential opportunities, the study investigated the planning and implementation procedures of NTD, as well as the challenges encountered, with possible suggestions to address the challenges. The study finally explored the opportunities to integrate health interventions into the extant structure, drivers, and barriers to the integration of public health intervention into the NTD structure, adapting the WHO health system framework.

### Understanding the working modalities of NTD structure in Yobe and Ebonyi states

The first objective of this study was to delve into the intricate workings of the NTD structure and its approach to implementing the MAM (Mass Administration of Medication) activity in selected Nigerian states. Thus, aligning with this critical objective, the study findings gleaned from insightful interviews with the study participants were expounded upon, covering four pivotal thematic areas: planning and coordination, implementation strategy, challenges encountered during implementation, and proposed mitigation strategies. Insights from this study shed light on the complexities and nuances of the NTD structure’s operational approach, revealing valuable lessons and potential remedies for addressing challenges in the implementation of MAM activities in the selected states.

### Planning and coordination

The results of the study revealed a robust and structured approach by the NTD committee in both states, with two layers of meetings conducted to reflect on past experiences from previous MAM activities and to strategically plan for upcoming MAM activities or other programs. The study participants emphasized the significance of these meetings, as expressed in their statements, underscoring the proactive and thoughtful approach taken by the NTD committee in ensuring the effective implementation of MAM activities. This was evident in the statement of the study participants who expressed that:

*In planning, we start with an annual review meeting because, for each year, we start from the past year and look for a way forward then after reviewing we now fix a strategic planning meeting that is the place where we put in place some gaps in years back some un-close gaps we do it and all the stakeholders are in the meeting even the LGAs and their HOD are in the planning meeting. During the meeting, we discuss our interventions and the CDDs, and their activities.* IDI/Ebonyi/SNTDC/2022.

*We have a structure. Even at the LGA level, we report to the state. So, we begin the year with a meeting with the state NTD, after which we also meet at the LGA level to discuss the previous year, and plan for the new year.* IDI/Yobe/LNTDC/2022.

This suggests a proper coordinating structure for the NTD in both states, as there was a clear coordinating structure. For both states, the LGA NTD does not work independently from the state, hence, NTD programs emanate from the state level and take courses in the LGAs. When further asked about the channel of coordination for the NTD, there was a consensus across the state and LGA regarding the structure.

*The LGA level report to us, at the state, and we report to the federal.* IDI/Yobe/SNTDC/2022.

### Implementation strategies of MAM in Yobe and Ebonyi states

Our results showed NTD implementation strategies were put in place across all relevant levels of stakeholders involved in the MAM routine. On that note, the study requested participants to share the strategy adopted to best reach members of the communities. Participants enunciated as follows:

#### Institutional support

*We use community mobilization strategy. We go to have meetings with the village heads, community leaders, traditional leaders that is how we normally do it and before we will finish doing that the message has gotten to the grassroots and as far as they know that something like this is coming in their domain and they are the ones asked to bring the CDDs you see our work is made easy, nobody will refuse, in fact, there was a place we went to and somebody refused that he will not or that they will not collect the drugs or that he will not be treated, we said okay and we made a call to the coordinator of the development center immediately he the person later agreed.* IDI/Yobe/LNTDC/2022.

*Immediately drug is given to us, we message the town crier to announce it to the people of the community and we provide the date for the drug to be given to them and the place that we normally stay to share the drugs like a playground. So on the day that it is given to them to come and collect the drug, they normally come, the town crier also tell them when they are coming they should also come with their water so that immediately the drug is given to you, you will use the water that you came with and swallow the drug and after that day we normally move house to house to give them the drug because some of them that did not participate in the first day, some who went to work then we will now move to their house to give them the medicine.* FGD/Ivo LGA/Ebonyi/2022.

*The way that we reach them, is that we inform them that we are about to give them Mectizan to make the announcement in our church, Catholic church, several churches. When we make the announcement, we do not spend any money, we simply just walk to their house very early in the morning so that they will take the Mectizan before they eat in the morning, and we tell them not to do any hard job for that day.* FGD/Ebonyi LGA/Ebonyi/2022.

This reveals that Advocacy Communication and Social Mobilization (ACSM) are fundamental approaches to the mass administration of drugs in rural Nigerian communities. In essence, buy—in is obtained from community leaders, who are in the best position to convince the members of their respective communities of the importance of the drugs being administered to the affected persons, as well as the implication of the drug administration to the population health of the community itself. Aside from ACSM, the CDDs also devise strategies to ease the process of house-to-house drug administration by using a town crier within the community to communicate a specific venue to members of the community that will be available at the specified time. Additionally, the town criers are used to foster adherence to drug usage on the part of the community residents, by informing community members to come along to the agreed venue with drinkable water, to ensure that they take the drug while they are with the CDDs. When drugs arrive in a community, CDDs commonly use religious centers as a means of reaching out to the people and forewarning community members of their approaching house-to-house visits. The CDDs engage in this activity as early as possible to avoid missing people who would be going out to fetch their daily bread, such as the farmers.

#### Access to means of transportation

Furthermore, another fundamental strategy adopted in the implementation of MAM is the use of transportation means to ease the implementation process. To this end, the participants emphasized that:

*The issues of transportation of logistics to some catchment areas because there are some nomadic settlement or area which requires more effort to reach out to them. So, what we used to do before was to take a bicycle, but it was not working well, so now we use a motorcycle so that we can get to those places.* FGD/Machina LGA/Yobe/2022.

*Some places are very far apart, so we used to use bicycles, but we now use motorcycles. We however want them to be adding to our transport because we are always buying petrol and it is now expensive.* FGD/Nangere LGA/Yobe/2022.

This suggests that in some states across the country, such as Yobe state in this study, certain settlements are far from other settlements, and CDDs may find it difficult to walk or move between them on foot. Therefore, it becomes very critical to get a means of transportation that would ease the efforts of reaching out to isolated communities. This means of transport may not necessarily be limited to road transport, but also include other means of transport like boat or canoe for communities that are separated by water, as we have in some parts of Nigeria such as Bayelsa.

### Challenges encountered during routine MAM implementation

#### Lack of incentives

In furtherance, the study participants were asked to highlight some of the challenges encountered while implementing the MAM activity in their respective states. Findings from the study revealed that the challenges reported to have been experienced more by the CDDs were greater than those experienced by the NTD coordinators at the state or LGA levels. However, the challenge of incentives was fundamental and raised worries among not only the CDDs but also the upper hierarchy within the NTD structure in each state. This has shown that there is a high attrition rate among CDDs, and this can be attributed to poor remuneration. Although the CDDs are a network of volunteers in the communities, they believe that they deserve better remuneration. This could be because of the stress they experience from the task, and as such they consider the stipends offered to them as meager and would barely help them in meeting their needs, particularly because they put in a significant amount of their time to distribute these drugs in the community.

*Our major challenge is that the CDDs incentives are small, and they have been complaining no matter the kind of preaching we have been giving to them, so that is the challenge, and this results in CDD attrition, to the extent that we have over 50 % attrition yearly.* IDI/Yobe/SNTDC/2022.

*The challenges, number one, we are not properly renumerated, I mean the payment is too small, how much do they pay you and how much do you want to be paid, they pay us just 3,000 Naira and all this one, even the old CDD that have been doing is the same amount since long time ago, so it’s not just good for the work.* FGD/Ezza South LGA/Ebonyi/2022.

*The major problem is money. What we are being paid is small, compared to what we do. We cover long distances just to administer these drugs, and then they pay us a meager amount.* FGD/Nangere LGA/Yobe/2022.

#### Inadequate equipment

Nevertheless, the challenges of the CDDs are not limited to remuneration but also extend to other issues. This suggests that the CDDs found the unavailability of weighing scales and other equipment challenging because they believed that this could lead to a breach of trust between the CDDs and the community members, particularly those who have experienced the use of body mass index (BMI)scales as well as stadiometers, used in measuring the individual’s height. The result also showed that the coordinators are aware of the unavailability of some useful equipment for the CDDs.

*The equipment for the work is not complete, I was going through their manual and I discovered that there should be provision for weighing balance because sometimes you will not find the stick and other things you use to measure the height, and some people are more conversant with weighing balance than that measuring stick, so those things should be put into consideration, there should be weighing balance and the stick so that anyone you are more conversant with you uses it so that you make sure you administer the drug appropriately.* IDI/Ebonyi/SNTDC/2022.

*The CDDs are not adequately equipped. There are some instruments that they should have that they do not have.* IDI/Yobe/LNTDC/2022.

#### Low acceptance

The level of acceptance by most households is still relatively low. While some are well receptive others are not. This suggests that CDDs experience mockery and rejection in many cases, and this can be attributed to the current economic situation in the country. This could dampen the morale of CDDs that experience such situations and could potentially result in the observed attrition in the numbers of CDDs.

*Some people make jest of us and outrightly chase us away. Sometimes when you enter someone’s compound, immediately you just step your feet in someone’s compound, he will just chase you out and tell you that you people should go and bring money and share and not be giving them drugs anytime. Other people that know the importance of the drug will accept us freely and will accept the drug.* FGD/Ivo LGA/Ebonyi/2022.

#### Insecurity

Another identified issue was insecurity, and this was generally reported by the CDDs in Yobe state. This connotes that insecurity is a bane to the successful implementation of MAM activity in Yobe State. However, such an issue is not limited to only Yobe state, as there exist insecurity issues in several states in the country, particularly in the Northeastern part of Nigeria. This, therefore, calls for urgent redress on the part of the government at the state and federal levels.

*There is an improvement only that the challenge that we are having here in Yunusari, is that there are partial insecurity issues though that has not stopped us from carrying out the program.* FGD/Yunusari LGA/Yobe/2022.

#### Unavailability of drugs

One of the challenges reported to have posed a threat to the implementation of MAM was the unavailability of drugs. The unavailability of drugs poses a serious impediment to not only the distribution of drugs in the communities but also the health of those that are affected by one form of NTD or the other, as well as the population health of the communities where people are affected reside. In other words, the drugs are not only of a positive effect on those who are taking the drug but also have indirect effects on other people in the communities. The drug helps to foster the extant social relationship in the communities among the people and subsequently reduces the fear of transmission and infection of disease from others because of the relationship shared.

*There are times when drugs are not available, and that is a big challenge.* KII/Ebonyi/LNTDC/2022.

*Sometimes, drugs are not available, so we wait till when drugs are available before we go out. But the people in the community, when they see us, they ask us when we are going to give them drugs.* FGD/Nangere LGA/Yobe/2022.

*Another major challenge is the unavailability of drugs, when they are not available, we sit and do not do anything. We wait for our LGA coordinators to inform us when drugs will be available.* FGD/Ivo LGA/Ebonyi/2022.

### Participants suggestions to identified challenges

#### Increase remuneration

The participants were asked to suggest possible mitigants to the various challenges experienced while implementing the MAM activity in the selected states. Participants across all levels shared similar opinions on the following: increased stipends and the regular availability of drugs.

*If the people working especially the CCDs can be well paid so they put more effort, this time around, especially as there is hunger and no food, you cannot do work without food, because it will affect your morale.* KII/Yobe/LNTDC/2022.

*The government should improve the allowance of CDD members, it is too poor and again the chiefs do not cooperate they do not even add Kobo to what we are given even when they visit them they do not even value us as good members of society.* FGD/Ebonyi LGA/Ebonyi /2022.

#### Consistent availability of drugs

On the constant availability of drugs, both CDDs and the NTD coordinators across the LGA and states levels believed that the availability of drugs is key to the success of the program. In essence, the availability of drugs will ensure the timeliness of implementing the MAM activity and would also go a long way in easing the implementation process itself for the CDDs in the various communities they will be visiting.

*If drugs are constantly available, then some of our challenges have been solved*. KII/Ebonyi/SNTDC/2022.

### Sensitization

Another recommendation to address the extant challenges is sensitization. This connotes that sensitization in communities can never be enough because residents in communities need to constantly be in the know of how things are with reference to the leadership of these communities because they would be able to convince their subjects. However, campaign and awareness structures should equally be put in place through key channels that would be able to increase people’s enlightenment. The provision of transportation is another proposed mitigation to the challenges.

*Then we are asking the government and other stakeholders to be more involved in creating awareness to people to make them know that these drugs are very important, they prevent diseases like river blindness, and skin diseases like scurvy so government should create awareness through the chiefs, the community chairmen to make people know that it is very important for people to take the drugs.* FGD/Nangere LGA/Yobe/2022.

### Ease of transportation

This suggests that the NTD coordinators at the LGA level have the interest of their NTD team at heart because they are soliciting a means of transportation for the team members who may be going to the LGAs for supervision. Such concern is equally applicable to Yobe State in this study and providing a means of transport would go a long way in ensuring the availability of all stakeholders that are required during the ACSM and implementation of the activity. A participant expressed that:

*We have had our meetings with the agency, so…. and we have made our report not only in this Ezza south but in all the local government in Ebonyi where we have our platforms and meetings as coordinators when we go for state meetings we table our challenges and in fact, one of the challenges when the pressure was much on them they gave the coordinators motorbikes like I have my own bike now all the thirteen LGA were given motorbikes. The coordinators will be able to supervise those areas, but the team, like the local government areas team if all things are equal, they should also have transportation means.* KII/Ebonyi/LNTDC/2022.

### Enablers of integrating health interventions into the NTD structure

#### Availability of funding

The study equally attempted to understand the participants’ perceptions of the enablers of integrating other health interventions into the NTD structure. Findings from the study participants revealed that across all levels, the participants believe that increased availability of finances would play a crucial role in the integration of other health interventions into the NTD structure. This suggests that the availability of finance is very important to the integration of health intervention into the NTD structure. The perception held at the state level may be associated with their dealings with several complaints from the CDDs through the LGA NTD coordinators. Their perception may equally be premised on the fact that while implementing the routine MAM activity, certain gaps might have been identified that require funding to be resolved. Essentially, a payment structure will serve as a form of motivation to the CDDs to do their work and do it excellently such that the upper hierarchy in the NTD structure is satisfied with the outcome of their endeavors.

*The potential driver I think is finance because people will want to be paid.* KII/Yobe/SNTDC/2022.

*The NTD structure, especially for the CDDs, needs a proper budget, especially to take care of finances and welfare. If the CDDs are properly remunerated and given permanent staff status with a proper payment structure, then it will be great.* KII/Ebonyi/SNTDC/2022.

*We need proper welfare for the CDDs because what attracts them is that they are given something after. If there is a proper means of welfare, then they would be encouraged to stay on, that way, we will reduce the attrition rate which is currently very high.* KII/Yobe/LNTDC/2022.

*The Need for Manpower.*

In furtherance, study participants also shared the perception that human resources are also core to the integration of other health interventions. This connotes that for the NTD coordinators, the efforts of the CDDs are well recognized within the communities, however, the integration of other health interventions into the NTD program would require that more CDDs are engaged, and the pattern of engaging CDDs to play roles and carry out responsibilities has been significantly beneficial to NTD program, as residents of those communities are familiar with the faces of the people who have administered drugs to them. This process was also recommended to foster the easy implementation of any other health intervention that may be integrated into the NTD structure. Additionally, CDDs believe in their competency and capacity to get the work done whenever such is required of them. Although integration may be significantly plausible, it would nevertheless require training of the CDDs before deploying them to their various communities to conduct any form of health intervention that may be required of them.

*Another thing that I will say is that there is a need for extra manpower, if you want to integrate other interventions, to make it easy, and it is better they are also from the communities.* KII/Yobe/LNTDC/2022.

*I’m of the opinion that government should use CDD workers in some of their health programs, like immunization, sharing of net, and numbering of houses* etc. *There should also be more people, that is, the CDD.* FGD/Ezza South/Ebonyi/CDD/2022.

### Possible challenges of integrating health interventions into the NTD structure

There is no gainsaying that healthcare integration (such as a horizontal intervention approach) is a major contributor to the growth of population health, through improved access to healthcare services. However, the integration or triangulation of health programs is not immune to challenges, and as such the study sought to understand the participants’ perceptions of the possible challenges that may impede the integration of other health interventions into the NTD structure. Fundamental to this is the lack of financial incentives, as such, participants emphasized that;

*Sometimes, financial aspect, because this time around, there is hunger, you cannot expect someone going from house to house to do work without taking food, so if they are giving reasonable money, they will do it very well.* KII/Yobe/SNTDC/2022.

Another participant noted that: *The only thing that will affect integrating health interventions into NTD is if they fail to pay us because with the situation of things, we are in now, everybody needs what to eat anyway that’s the thing.* KII/Ebonyi/LNTDC/2022.

This suggests that avoidance of monetary payments to CDDs will significantly affect the success of any health intervention leveraging the NTD structure. This is because the CDDs not only consider this as a volunteering event but are also expectant of financial motivation or benefit that would come with this, as this would help them address their own personal and family needs. Participants were also of the perception that lack of cooperation between partners could negatively affect integrating other health interventions into the Nigerian NTD structure.

*The partners that are piloting the NTD activities I do not know if they will agree to incorporate other health interventions into their own.* KII/Ebonyi/SNTDC/2022.

This suggests that partners’ buy-in is equally crucial to successful integration. Essentially, as has been discussed, partners may refuse to play their part in funding if they feel or believe that the government (who is to act in the capacity of a bridge between two or more health programs) has refused to carry them along in the integration process, and that may subsequently affect the activities of the NTD program in the state or the country at large. Work overburden was another barrier to integration pointed out by the study participants.

*The only challenge I will say is everything lies on those who will do the work that is the only challenge that will hinder the integration, there was a time when the rollback malaria started, and they integrated with the NTD, in fact everything they were doing, it was the CDD doing it, people became tired, and they were complaining. In fact, the first exercise that was done, the payment of the workers was handed to me by the project coordinator, and they gave me over a million to pay the workers. Eventually, it became so much work for us, and after sometimes they drop the CDDs, which was not good because they did a lot of work.* KII/Ebonyi/LNTDC/2022.

This suggests that integration of other health interventions may require more effort and endeavors on the part of the CDDs which they may not be used to, and as such may be considered an overburden of work compared to what they were used to. Generally, health workers tend to experience burnout when they are overwhelmed with work, and this can also apply to CDDs as well. It is therefore important to consider the workload that will be involved in the integration of other health interventions and address possible gaps that may be identified before the commencement of the project.

## Discussion

As shown from this study, the NTD structure in Ebonyi and Yobe states has efficient structure and effective implementation processes, with appropriate measures in place for the MAM activity within the states. Findings from our study objectives established the following five of the six components of the WHO health systems framework; Service Delivery (demand generation and social mobilization), Health Workforce (need for manpower), Access to Essential Medicines (Mass Administration of Medicines), Financing (improved welfare, increased stipends, and motivation), and Leadership and Governance (planning and coordination). This has aided the achievements of previous program implementations and has created the avenue to integrate other health interventions. Such had been reported by Means et al. (21) with relative effectiveness in the NTD program activities highlighted. However, findings from our study identified that there is a gap in structured data reporting on the NTD program in Ebonyi and Yobe states, as recommended in the WHO health systems framework. We perceived that this might have hindered the extent to which insights into the program’s success are accessed especially for further decision-making.

Based on the working modalities of the NTD structure, two levels of planning meetings are held within the NTD structure wherein the teams evaluate the previously conducted activity and try to address the gaps found in their evaluation through the development of a new plan for the implementation of the next activity. This strategy is laudable and recommendable because, through constant iterations, it significantly improves and solidifies the implementation plan such that it eventually becomes a point of reference for the implementation of similar programs. It equally promotes the sustainability of the program being implemented because structural adjustments made to the plan are premised on the implementation experiences.

The NTD program adopts various strategies to get their work done. Fundamental to the strategies adopted is ACSM wherein all stakeholders at the community levels including the community leaders and religious leaders are met, to discuss the activity to be conducted in the community and the buy-in of these stakeholders is obtained. This is important because it helps to make the implementation easier as the stakeholders at the community level play an integral role in convincing residents of the communities in the localities where this activity will take place. Building a stakeholder relationship and involving the public has also been noted to potentially help in reaching NTD outcomes (22). Therefore, conducting the activity is premised on the trust that exists between the community leaders and members of the community. Also, the CDDs adopt several communication channels to reach out to the community members to keep them informed ahead of their visit to their community to administer the drugs. Notable of these channels is the town crier and the religious centers. Town criers have been reported as agents of passing information to the public on health interventions such as the use of non-pharmaceutical interventions (like a face mask, and hand sanitizer) during epidemics and pandemics (23, 24). Oku et al. (10) also noted that adopting community and religious leaders as a communication strategy hasten the process of administration and information passage to the community members. Additionally, these channels are relevant to the socio-cultural values and settings that exist in the communities that are visited. All of these alluded to the proper planning and coordinating structure of the NTD in Ebonyi and Yobe states.

The crop of challenges however identified in the NTD structure during this study includes lack of a means of transportation, shortage of equipment and drugs, shortage of manpower, attrition of the CDDs, as well as lack of motivation of the CDDs which led to a high attrition rate. These challenges have been noted to affect the health system, and as such could hamper the integration of health interventions into the NTD structure. Since a healthy health system is predicated on having motivated health professionals, a well-kept infrastructure, and a consistent supply of medications and technologies supported by sufficient money, robust health plans, and evidence-based policies (8), most of these challenges as suggested by the participants would be mitigated with better funding, a proper budget line for NTD structure and more involvement of government and other partners alike. Also highlighted was the addition of more CDDs which would help to reduce the excessive workload which has been reported to potentially affect the productivity of the CDDS, thus affecting the overall output of the NTD program and the integrated health intervention. Excess workload and overburdening of health workers have been reported as a barrier to integration in primary healthcare settings (25–27). A lack of cooperation between intervention funders and the NTD structure may also significantly affect the potential integration of health interventions into the NTD structure as highlighted by the study participants, as such, lack of cooperation represents a *clog in the wheel* to integrating other health interventions into the NTD structure. Since intra-sectoral and multi-sectoral partnerships are based on cooperation and trust to foster collaboration, funders, NTD structure, and other stakeholders must synergize to achieve the goal of any intervention that is to be implemented.

## Conclusion

Based on the findings from the study, the integration of public health interventions into the NTD structure would be a great feat as future health interventions would benefit from the existing structure of the NTD program. The modalities and the processes that are in place for the NTD program have not only proven that the implementation of health interventions can be efficient but can also give room for other interventions to be incorporated. Essentially, this study alludes to the fact that an appropriate and organized structural system for public health intervention provides opportunities for not only integrating future interventions but also increasing the services of the existing health intervention, hence, future public health interventions can be integrated into the NTD program in Nigeria.

Although findings from our study supported five of the six WHO health system framework, we realized that there is a gap in the NTD structure relating to the health information system. There seems to be an absence of a structured data reporting system that could aid decision-making and provide insight on distribution coverage. This study therefore recommends the need for further studies assessing health integration to consider this component.

## Data availability statement

The original contributions presented in the study are included in the article/supplementary material, further inquiries can be directed to the corresponding author.

## Ethics statement

The studies involving humans were approved by National Health Research Ethics Committee, Nigeria (NHREC). The studies were conducted in accordance with the local legislation and institutional requirements. The participants provided their written informed consent to participate in this study.

## Author contributions

SA: Conceptualization, Methodology, Supervision, Writing – review & editing. SS: Conceptualization, Investigation, Project administration, Supervision, Writing – review & editing. AJ: Conceptualization, Methodology, Project administration, Supervision, Writing – review & editing. SI: Conceptualization, Methodology, Writing – original draft, Formal analysis. OA: Supervision, Writing – review & editing, Writing – original draft. JD: Data curation, Investigation, Methodology, Project administration, Supervision, Writing – original draft. VD: Methodology, Project administration, Supervision, Writing – review & editing. OD: Data curation, Methodology, Supervision, Writing – original draft.
